# Identification and Expression Analysis of the Nucleotidyl Transferase Protein (NTP) Family in Soybean (*Glycine max*) under Various Abiotic Stresses

**DOI:** 10.3390/ijms25021115

**Published:** 2024-01-17

**Authors:** Liqing Kang, Changgen Li, Aokang Qin, Zehui Liu, Xuanyue Li, Liming Zeng, Hongyang Yu, Yihua Wang, Jianbo Song, Rongrong Chen

**Affiliations:** 1 College of Bioscience and Bioengineering, Jiangxi Agricultural University, Nanchang 330045, China; kanglq2023@shanghaitech.edu.cn (L.K.); lichanggen123@stu.jxau.edu.cn (C.L.); 15296718993@163.com (A.Q.); liuzehui125@163.com (Z.L.); 15870780929@163.com (X.L.); zlm@jxau.edu.cn (L.Z.); hongyangyu@jxau.edu.cn (H.Y.); wwyihua@126.com (Y.W.); 2 School of Life Science and Technology, ShanghaiTech University, Shanghai 201210, China

**Keywords:** nucleotidyl transferase protein (NTP), *Glycine max*, abiotic stress, RNA-seq, gene expression, subcellular localization

## Abstract

Nucleotidyl transferases (NTPs) are common transferases in eukaryotes and play a crucial role in nucleotide modifications at the 3’ end of RNA. In plants, NTPs can regulate RNA stability by influencing 3’ end modifications, which in turn affect plant growth, development, stress responses, and disease resistance. Although the functions of NTP family members have been extensively studied in *Arabidopsis*, rice, and maize, there is limited knowledge about NTP genes in soybeans. In this study, we identified 16 members of the NTP family in soybeans, including two subfamilies (G1 and G2) with distinct secondary structures, conserved motifs, and domain distributions at the protein level. Evolutionary analysis of genes in the NTP family across multiple species and gene collinearity analysis revealed a relatively conserved evolutionary pattern. Analysis of the tertiary structure of the proteins showed that NTPs have three conserved aspartic acids that bind together to form a possible active site. Tissue-specific expression analysis indicated that some NTP genes exhibit tissue-specific expression, likely due to their specific functions. Stress expression analysis showed significant differences in the expression levels of NTP genes under high salt, drought, and cold stress. Additionally, RNA-seq analysis of soybean plants subjected to salt and drought stress further confirmed the association of soybean NTP genes with abiotic stress responses. Subcellular localization experiments revealed that GmNTP2 and GmNTP14, which likely have similar functions to HESO1 and URT1, are located in the nucleus. These research findings provide a foundation for further investigations into the functions of NTP family genes in soybeans.

## 1. Introduction

Nucleotidyl transferase proteins (NTPs) are a type of proteinase with terminal nucleotide transferase activity that catalyze nucleotide transfer to the ends of RNAs, especially small RNAs [1]. In eukaryotes, NTPs have the ability to catalyze the addition of non-templated nucleotides at the 3’ end of small RNAs, which can regulate their stability [2,3]. Genetic evidence from plants and animals has shown that small RNAs play important roles in growth, development, and stress responses [4,5,6,7]. Some NTPs affect small RNAs by both the direct addition of nucleotides at the 3’ end and indirect effects on their abundance [8]. The modifications of small RNA by NTPs are mainly divided into adenylation, uridylation, cytidylation, guanylation, and compound modification. Uridylation caused by some NTPs can trigger the degradation of miRNAs and siRNAs, leading to significant changes in growth and development [8,9]. In contrast, the adenylated miRNAs contribute to their stability in in vitro degradation systems and in animals [10,11]. In addition, a number of cytidylations, guanylations, and compound modifications have been found at the 3’ end of small RNAs in plants and animals, but their origin and function remain unclear; they are probably caused by other NTP members [12,13]. Considering the important modification effect of NTPs on small RNAs, it is necessary to identify and investigate NTPs in different species.

*NTP* genes have been studied in multiple species, such as human, mouse, yeast, *Chlamydomonas*, *Drosophila*, *Caenorhabditis elegans*, *Arabidopsis*, rice, and maize [9,14,15,16,17]. In humans, nucleotide transferases are divided into seven types based on the HUGO-approved gene names [9,14,15,16,17]. Among them, TUT1, TUT4, and TUT7 have uracil ribonucleotide specificity. TUT1, TENT2, TENT4A, TENT4B, and MTPAP have adenine ribonucleotide specificity. TENT2 may also have specificity for uracil and guanine ribonucleotides. TUT1 adds a longer polyU tail at the 3′ end of U6 snRNA, which recruits USB1 removing uridines and forming 2′,3′-cyclic phosphate in the mature process [18]. In addition, TUT1 affects the addition of 3’ end nucleotides to specific miRNAs to regulate miRNA abundance [3]. The highly similar gene structures of TUT4 and TUT7 arise from a gene duplication event [19]. TUT4 adds a short polyU tail to pre-let-7 to cooperate with LIN28A, preventing Dicer cleavage and promoting subsequent degradation [14]. TUT4/7 oligo-uridylates pre-let-7 by a Lin28-dependent mechanism and blocks Dicer processing to repress the expression of let-7, regulating cell differentiation and proliferation [20,21]. GLD-2 adenylates the 3′ end of miR-122 to promote its stability in mouse liver cells and mono-uridylates some miRNAs to promote their stability in human fibroblasts [22]. In the fission yeast Schizosaccharomyces, Cid1 uridylates the 3′ end of posttranscriptional actin mRNAs in S-phase arrest [15]. In *Chlamydomonas*, MUT68 adds non-templated uridine at the 3′ end of mature miRNAs and siRNAs, and synergizes with PRP6 to promote their degradation [9]. CG1091 is a specific uridine transferase in *Drosophila*, which mainly adds uridine to the 3’ end of mirtron hairpins and, to a lesser extent, to canonical pre-miRNAs [16]. CDE-1 is a nucleotide transfer enzyme found in nematodes that can add nontemplate uridine to the siRNA bound to CSR-1 [17]. Above all, the mechanism of NTPs has been extensively studied in animals, providing important clues for the investigation of NTPs in other species.

Compared to animals, there are fewer reports of NTPs modifying small RNAs in plants, especially in terms of stress response. Currently, NTP family genes have been identified in the genomes of various plants, including *Arabidopsis*, rice, and maize, with a total of 10, 13, and 24 members, respectively [1,23,24]. *HESO1* (*AtNTP1*) is the first NTP family gene found in plants, as the inhibitor of the methyltransferase HUA ENHANCER1 (HEN1) in miRNA biosynthesis, which can add non-templated uridine at the 3’ end of the unmethylated mature miRNAs and pre-miRNAs in *Arabidopsis*, effecting their degradation and promoting further processing of pre-miRNAs [8]. In a recent report, cytidylation was observed in pre-miRNAs in *Arabidopsis*, which was caused by HESO1 and NTP6/NTP7 [12]. HESO1 was also found to be involved in the uridylation of the 3′ end of Pol IV/RDR2-dependent dsRNAs (P4RNAs) in *Arabidopsis*, contributing to its stability [25]. In addition, URT1 (AtNTP3) is complementary to HESO1, and they work sequentially and collaboratively to uridylate different forms of the same miRNA in vivo [26]. URT1 is the main terminal uridylyl transferase (TUTase) involved in mRNA uridylation (70–80%), compared to HESO1, and directly interacts with DECAPPING 5 (DCP5) to prevent the accumulation of excessive deadenylated mRNAs and to decrease the biogenesis of spurious siRNAs [27]. However, HESO1 is the major TUTase to uridylate the 5’ fragments produced by RISC-cleaved mRNAs [28]. To our knowledge, the mechanism of terminal modification and the NTP functions remain largely uninvestigated in plant species other than *Arabidopsis*. Previous research has demonstrated that NTPs can be involved in the terminal modification of RNAs to regulate their stability during various plant growth and development processes [8,29]. The conserved core domains may allow certain NTPs to perform the same or similar functions as HESO1 and URT1 in other plants.

RNA-seq and experimental analyses have shown that the expression of the NTP family changes significantly under abiotic stress [1,24]. It is likely to affect the stability of RNA in response to stress by regulating the expression of NTPs. In rice, several *OsNTP* genes were specifically expressed at particular developmental stages and clearly induced by salt, drought, heat, cold, or ABA treatments [1]. For example, *OsNTP5/6/7/8* were induced by salt stress, *OsNTP1*/2/4/9/13 were upregulated under drought conditions, *OsNTP4*/5/6 were induced by ABA treatment, *OsNTP3/4/5/12/13* were downregulated by cold treatment, and *OsNTP3/4/6/8/11* were induced by heat stress. In maize, *ZmNTP1/5/6/9/10/11/12/16/17/18/19/20/21/22* were significantly downregulated in aboveground tissues under drought stress; the only upregulated genes were *ZmNTP23* and *ZmNTP24*, although *ZmNTP2/3/4/8/9/17/20/22/23/24* were induced in the roots [24]. The expression level of *ZmNTP* genes did not change significantly in the aboveground tissue under salt stress, but *ZmNTP4/8/9/22* were significantly upregulated in the roots and *ZmNTP2/3/7/11/13/15/19/21* were significantly downregulated. In conclusion, the *NTP* genes may play an important role in plant stress response, but there is a lack of genetic evidence.

NTPs plays a crucial role in modifying nucleotides at the 3’ end of plant RNA in plants, which affects the stability of RNA in plants, especially small RNA. Small RNAs in plants are highly significant for aspects such as growth, development, stress resistance, and disease resistance. In recent years, there have been many reports on the functions of NTP family members and their key genes in *Arabidopsis*, rice, and maize [24]. However, there is a lack of relevant research in soybean, an important oil crop. Therefore, we selected 16 *NTP* genes from the soybean genome and analyzed their distinct characteristics at both the protein and gene levels. We further investigated the expression of GmNTP genes in various tissues and their responses to different abiotic stresses. These results are expected to provide basic information for future functional studies of GmNTPs.

## 2. Results

### 2.1. Identification and Characterization of GmNTP Family Genes

To identify NTP family genes, we used the NTP_transf_2 domain (PF01909) to perform bioinformatics searches in the ‘Williams 82’ genome. Sixteen putative *GmNTP* genes were detected in the genome of soybean through bioinformatics analysis and were named GmNTP1 to GmNTP116 based on their order on the chromosomes (Table 1). According to conserved motifs and phylogenetic relationships, sixteen *GmNTP* genes were further classified into two subfamilies: G1 and G2. The molecular mass of the 16 NTP proteins ranged from 43.3 kDa to 170.6 kDa, and the average total length was approximately 785 amino acid residues, with most GmNTP proteins ranging from 600 to 800 amino acid residues. The isoelectric point (pI) of GmNTP family proteins is 6.70 on average, ranging from 5.3 to 9.68, and leaning towards neutrality and slight acidity. The variation in molecular weight and pI among GmNTP proteins suggests potential functional diversity within the family.

### 2.2. Phylogenetic Evolution and Chromosomal Localization of GmNTP Genes

**Figure 1:**
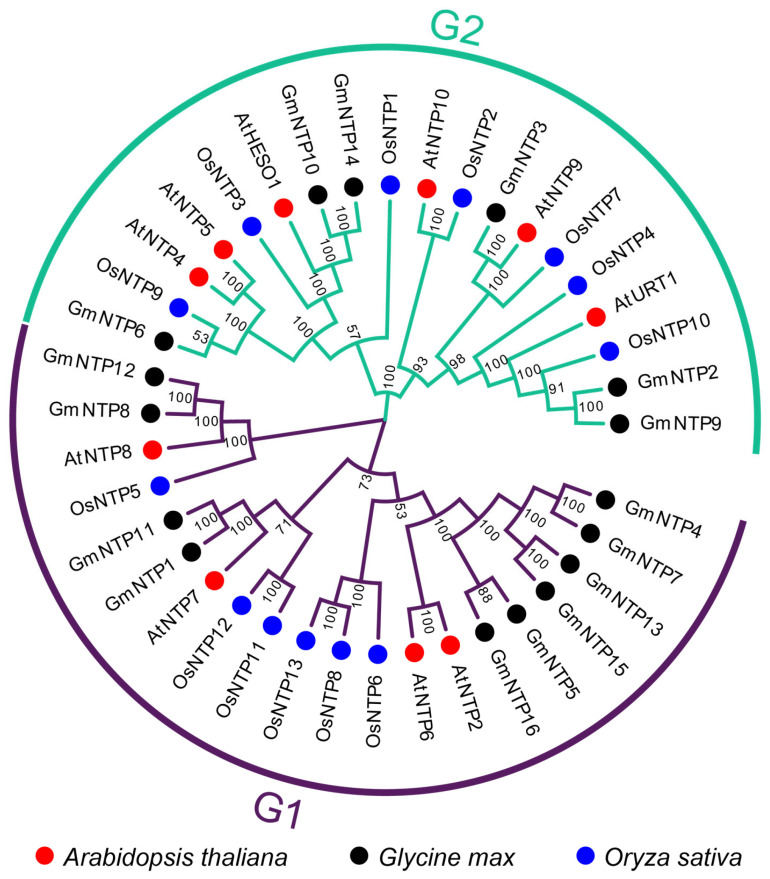
Phylogenetic analysis of GmNTP proteins. Red circles represent the NTP family members of *Arabidopsis thaliana*; black circles represent the NTP family members of *Glycine max*; blue circles represent the NTP family members of *Oryza sativa*. The resulting *NTP* genes were categorized into two distinct groups (G1/G2), G1 is distinguished by purple and G2 by green on the evolutionary tree..

A phylogenetic tree of GmNTPs was constructed and analyzed to assess the evolutionary relationship of *NTP* genes among *Arabidopsis*, rice, and soybean (Figure 1). The full-length NTP protein sequences were aligned, and a neighbor-joining (NJ) tree was constructed by MEGA7 using the bootstrap parameter to 1000. Based on the phylogenetic tree, NTP family members were divided into two branches. The G1 subfamily consisted of *GmNTP1*, *GmNTP4*, *GmNTP5*, *GmNTP7*, *GmNTP8*, *GmNTP11*, *GmNTP12*, *GmNTP13*, *GmNTP15*, and *GmNTP16*. The G2 subfamily includes *GmNTP2*, *GmNTP3*, *GmNTP6*, *GmNTP9*, *GmNTP10*, and *GmNTP14*. *GmNTP4*, *GmNTP5*, *GmNTP7*, *GmNTP13*, *GmNTP15*, and *GmNTP16* were homologous to *AtNTP2* and *AtNTP6*. *GmNTP1* and *GmNTP11* were homologous to *AtNTP7*. *GmNTP8* and *GmNTP12* were homologous to *AtNTP8* and *OsNTP5*. *GmNTP10* and *GmNTP14* were homologous to *AtHESO1* and *OsNTP3*. *GmNTP3* was homologous to *AtNTP9* and *OsNTP7*. *GmNTP2* and *GmNTP9* were homologous to *AtURT1* and *OsNTP10*.

**Figure 2:**
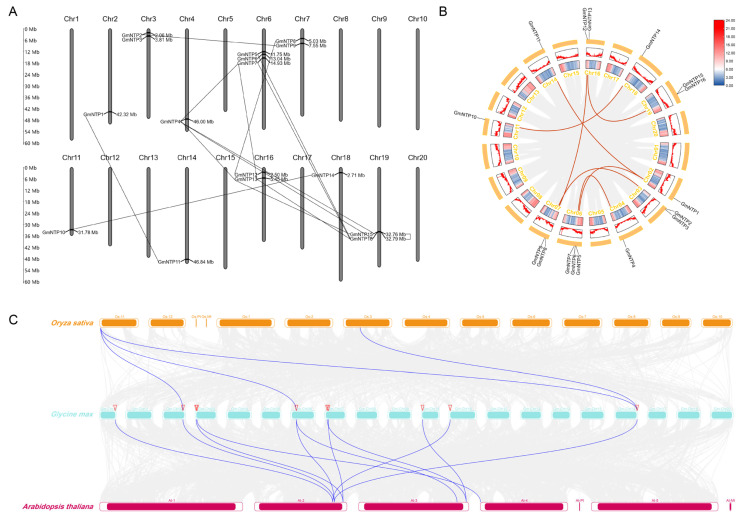
Chromosomal location and collinearity analysis of *GmNTP* in soybean. (**A**) Locations of *GmNTP* genes in twenty chromosomes of soybean. The protein sequences encoded by straight-line junction genes are more than 70% similar. (**B**) Genome-wide collinearity analysis of NTP in the soybean genome. Paralogous genes were linked by red lines represent. The inner circular heatmap represents gene density, red represents high density and blue represents low density. (**C**) Genome-wide collinearity analysis of *NTP* genes between the soybean (blue), *Arabidopsis* (red), and rice (yellow) genomes. Blue lines represent orthologous genes; red triangles represent different gene pairs.

To visualize the chromosomal localization of *GmNTP* genes, the physical positions of 16 *GmNTP* genes were extracted from the Williams 82 genome and are shown in the chromosomal map (Figure 2A). The 16 *GmNTP* genes were randomly distributed on soybean chromosomes. The *GmNTP* genes are mostly distributed on chromosome 6, with *GmNTP5*, *GmNTP6*, and *GmNTP7*, but half of the chromosomes have no *NTP* gene distribution. Furthermore, the *GmNTP* genes on each chromosome are positioned near the chromosome ends. The furthest distance between multiple *GmNTP* genes on the same chromosome does not exceed 3.5 Mb; moreover, they tend to cluster on the same side of the chromosome, which implies that they are likely derived from the common ancestor. Among them, the largest intergenic distance was observed between *GmNTP5* and *GmNTP7* on chromosome 3, with a gap of 3.18 Mb, whereas the shortest distance was found between *GmNTP15* and *GmNTP16* on chromosome 19. In addition, we found 15 gene pairs involved in gene duplication events by sequence alignment with the following criteria: (1) alignment of the sequences covering ≥80% of the longest gene and (2) sequence similarity ≥70% within the aligned region (Figure 2A) [30,31]. Among these duplicated genes, five pairs exhibited a genetic similarity greater than 90%: *GmNTP8/12*, *GmNTP1/11*, *GmNTP13/15*, *GmNTP2/9*, and *GmNTP10/14*. Finally, among the 16 *GmNTP* genes, only *GmNTP3* and *GmNTP6* do not have corresponding duplicated genes.

### 2.3. Collinearity Analysis and Evolutionary Relationship of NTP Genes

Collinearity analysis was performed to explore the evolutionary relationships among *NTP* genes in the soybean genome (Figure 2B,C and Table S3a). The analysis revealed several collinear *NTP* gene pairs among *GmNTP* genes. Specifically, *GmNTP1* showed collinearity with *GmNTP11* and *GmNTP5*. *GmNTP2* and *GmNTP9*, which are homologous to *AtURT1*, exhibited collinearity. Additionally, collinearity was observed between *GmNTP4/7*, *GmNTP8/12*, and *GmNTP8/12. GmNTP10* showed collinearity with *GmNTP14*, which is homologous to *AtHESO1*.

To explore collinearity relationships of *NTP* genes across multiple species, we conducted genome alignments between *Arabidopsis thaliana*, rice, and soybean (Figure 2C and Table S3b). The comparison of soybean and *Arabidopsis* genomes revealed the collinearity of *GmNTP1/5/11* and *AtNTP2. GmNTP5* also exhibits collinearity with *AtNTP7*. Similarly, *GmNTP2/9* exhibited collinearity with *AtURT1*; *GmNTP8/12* exhibited collinearity with *AtNTP8*; *GmNTP3* displayed collinearity with *AtNTP9*; and *GmNTP10/14* exhibited collinearity with *AtHESO1*. In the comparison of rice and soybean genomes, collinearity was observed between *GmNTP1* and *OsNTP11*. Moreover, *GmNTP5* exhibited collinearity with *OsNTP11*; *GmNTP11* exhibited collinearity with *OsNTP6/11*. The results further support that NTPs are conserved in different plants and may have similar functions in different plants.

To further explore the evolutionary relationships of NTP genes, we calculated the substitution ratio mutation (Ka/Ks) of these collinear gene pairs (Table S4). When Ka/Ks is less than 1, it suggests purifying selection, where the selection process counteracts mutations to preserve protein stability. Conversely, when Ka/Ks exceeds 1, positive selection is at play, signifying substantial mutations in genes that ultimately result in alterations to the encoded proteins [32]. The Ka/Ks value of the detected gene pairs ranges from 0.096 to 0.441, suggesting that purifying selection plays an important role in the evolution of NTP genes.

### 2.4. Conserved Motifs Analysis of NTP Genes

**Figure 3:**
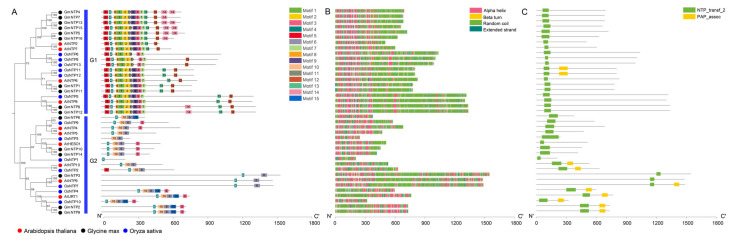
Conserved motifs, secondary structure, and conserved domain analysis of NTP proteins in soybean. (**A**) Conserved motifs of NTP proteins. Fifteen motifs are displayed in different colors. (**B**) Secondary structures of NTP proteins. Different colors represent different secondary structures. (**C**) Conserved domain of the NTP protein. Green represents the NTP_transf_2 domain, and yellow represents the PAP_assoc domain.

After all the *NTP* genes of the three species were analyzed online based on MEME, 15 conserved motifs of the NTP proteins were identified (Figure 3A and Figure S1). The G1 subfamily shares a conserved motif arrangement from the N-terminus to the C-terminus: Motif5, Motif4, Motif8, Motif1, Motif2, Motif11, Motif9, Motif3, Motif7, Motif13, and Motif12. This continuous conserved motif structure among G1 subfamily members suggests a high degree of structural similarity and potentially similar biological functions. The G2 subfamily features a conserved motif arrangement from the N-terminus to the C-terminus: Motif4, Motif10, Motif6, and Motif3. Notably, *GmNTP10/14* share a common motif arrangement and a high degree of evolutionary relationship with *AtHESO1*. Similarly, *GmNTP2*/9 share a common motif arrangement and a high degree of homology with *AtURT1.* These results indicate that these proteins exhibit similar functions. The core region of NTP_transf_2 corresponds to Motif 4 (GS--D-D). The conserved GS and D in Motif 4 are retained across all NTP genes, implying their functional significance in nucleotide transferase activity. *GmNTP3*, *AtNTP9*, and *OsNTP7* only possess Motif4 and Motif6, and they have long nonconserved sequences, distinct from other NTP family proteins, indicating a potential specific function for these three genes.

### 2.5. Secondary Structure and Conserved Domain Analysis of GmNTP

To further analyze the structural characteristics of the *GmNTP* genes, we predicted the distribution of the secondary structure by SOPMA tools (Figure 3B and Table S5). The secondary structure of the NTP family consists of a large number of alpha helices and random coils and a small number of beta turns. The G1 subfamily has a relatively conserved secondary structure, while the G2 subfamily has many patterns in the secondary structure distribution. In addition, we conducted a conserved domain analysis using the hmmscan tool, in which NTP_transf_2 was the conserved and core domain in the NTP family (Figure 3C and Table S6). Most of the NTP_transf_2 domain was located in the N-terminus of these NTP protein sequences, but the NTP_transf_2 domain of *GmNTP2/3/9* was closer to the C-terminus. Moreover, we identified the PAP_assoc domain located within 300 amino acids downstream of the NTP_transf_2 domain, which was identified as having polynucleotide adenylyltransferase activity. These PAP_assoc domains are predominantly found at or near the protein’s C-terminus.

### 2.6. Tertiary Structure Prediction and Analysis

**Figure 4:**
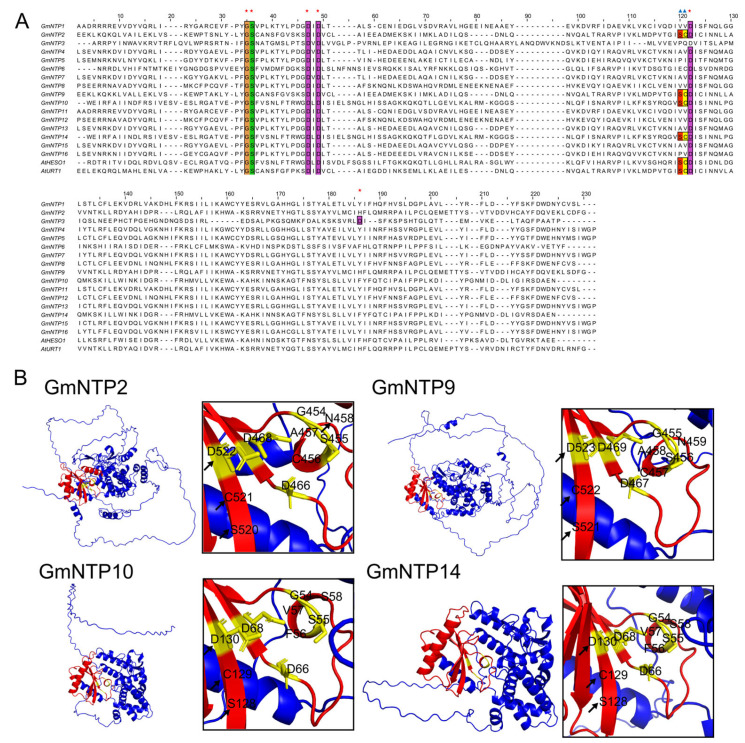
Multi sequence alignment and tertiary structure analysis of GmNTP proteins. (**A**) Conserved amino acid sequence analysis of the core domain of GmNTP family. The red pentagrams mark the conserved sites of NTP_transf_2 (PF01909) and the blue triangle mark the potential site with uridine function in plants. Different colors represents different amino acids. (**B**) Tertiary structure prediction and analysis of GmNTP2/9/10/14 proteins. The possible active centers formed by three conserved aspartates are shown in the detailed view. The arrow points to a conserved SCD structure.

To understand the active centers of NTP proteins, we constructed multiple sequence alignments using muscle and predicted their tertiary structures by Alphafold2 (Figure 4A,B). The results revealed that there were three conserved aspartic acids in 16 GmNTP proteins, which is consistent with previous experimental results for NTPs in animals and plants [33,34,35,36]. Although these three aspartic acids are several tens of amino acids apart in sequence, they bind together to form a catalytic aspartate triad in space (Figure S2). Previous studies have shown that UTP-Mg^2+^ binds to the first two aspartates of the TbRET2 by hydrogen bonds. The third conserved aspartate is close to the α-phosphate group of the UTP, and its O^δ1^ is hydrogen bonded to the UTP O^α1^ via a bridging water molecule [35]. This water position might be occupied by a second metal ion when it works, and the third conserved aspartate might be essential during the uridine monophosphate moiety transfer step. Interestingly, the third aspartic acid of HESO1 and URT1 forms a conserved SCD sequence with the two amino acids before it, which only exists in their homologous gene GmNTP2/9/10/14 (Figure 4B), which is unique to plant TUTase. It is possible that the SCD structure plays a significant role in the uridylation of small RNA at the 3’ end in plants.

### 2.7. Protein Interaction Networks and Gene Co-Expression Networks Analysis of GmNTP

**Figure 5:**
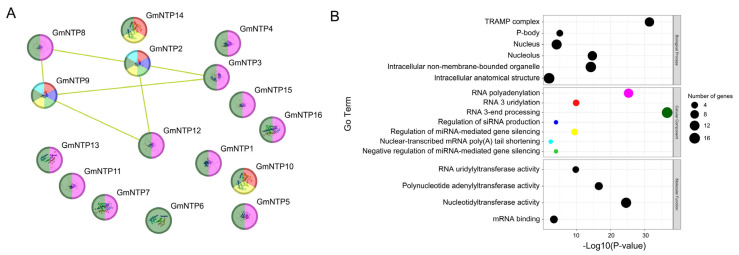
Protein interacting network and functional analysis of GmNTPs. (**A**) Protein interaction networks in GmNTPs. The lines represent possible interactions. The different color represent the different cellular component of the GO enrichment results such as green means those genes is involved in RNA 3-end processing (**B**) GO enrichment shown as dot bubbles. A total of 17 GO terms were significantly enriched, covering biological processes, cellular components, and molecular functions. The smaller the circle, the fewer the number of genes.

To further explore the relationships among GmNTP family members, we constructed the protein interaction network for GmNTP (Figure 5A). The interactions of most GmNTP proteins were not discovered. However, GmNTP2/9, homologous to URT1, showed potential interactions with GmNTP3/8/12. In addition, we also constructed interaction networks between GmNTP and other proteins (Figure S3). The proteins from the G1 subfamily, except for GmNTP8/12, were predicted to interact with the 3’,5’ RNA exonuclease activity of the HRDC domain-containing protein (K7L118). The G1 subfamily proteins and GmNTP3 were predicted to interact with the RNA helicase activity of the DSHCT domain-containing protein (A0A0R0H3J5). By combining this information with GO enrichment results (Figure 5B, Table S7), GmNTPs were found to be involved in RNA 3’ end processing and possess nucleotide transferase activity. They participate in the formation of the TRAMP complex, which is associated with nuclear surveillance, turnover of noncoding RNAs, and intergenic transcripts [37]. It is worth noting that GmNTPs, except for GmNTP2/6/9/10/14, were enriched in the function of RNA polyadenylation, which has never been reported in NTPs related to adenylation in plants.

To obtain more insights regarding the interactions of NTP genes with other genes in soybean, we constructed a gene co-expression network (Figure S4). Five pairs of genes were found to have coexpression relationships, which was likely due to their homology. Four genes were found to have coexpression relationships with other proteins. GmNTP5 and GmNTP16 were not found to be related to other genes. A total of 167 genes was found in the co-expression network of NTP genes; these were found to be involved in ubiquitin-mediated proteolysis (KEGG ID: gma04120), endocytosis (KEGG ID: gma04144), peroxisome (KEGG ID: gma04146), protein processing in endoplasmic reticulum (KEGG ID: gma04141), and fatty acid elongation (KEGG ID: gma00062). Among them, LOC100783831 (probable E3 ubiquitin-protein ligase LOG2), LOC100786561 (cullin-1), LOC100789233 (E3 ubiquitin-protein ligase UPL5), LOC100793682 (cullin-1), LOC100802630 (E3 ubiquitin-protein ligase UPL1-like), LOC100812184 (E3 ubiquitin-protein ligase UPL1), LOC100819012 (E3 ubiquitin-protein ligase UPL1) were identified as co-expressed genes involved in ubiquitin-mediated proteolysis. LOC100784075 (epidermal growth factor receptor substrate 15-like 1), LOC100793159 (intersectin-2), LOC100794495 (epidermal growth factor receptor substrate 15-like 1), and LOC100818930 (actin cytoskeleton-regulatory complex protein pan1) were identified as co-expressed genes involved in endocytosis. The co-expression results revealed the critical functions of NTP genes during plant growth.

### 2.8. Cis-Element Analysis and Gene Structure of GmNTP Genes

**Figure 6:**
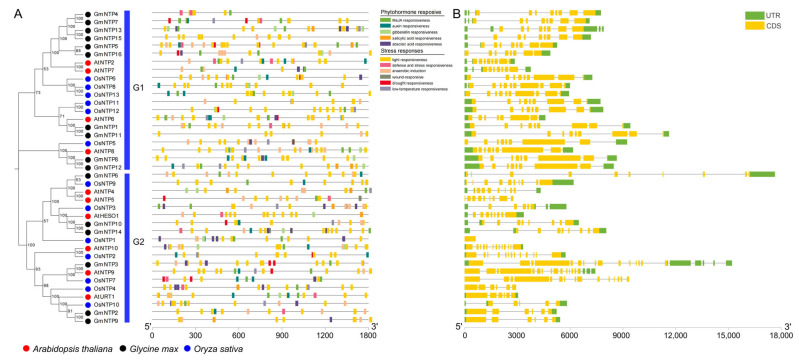
*Cis*-acting elements and gene structure analysis of *NTP* genes in soybean. (**A**) *Cis*-acting elements in the promotors of *NTP* genes. Different colors represent different *cis*-acting elements. (**B**) Gene structures of *NTP* genes. The yellow and green boxes represent the coding DNA sequence (CDS) and untranslated region (UTR), respectively.

*Cis*-acting elements are DNA sequences located upstream of the gene coding region that play a regulatory role by responding to various factors. The 1500 bp sequences upstream of all *GmNTP* genes were analyzed by the PlantCARE database, which found numerous *cis*-regulatory elements related to phytohormone response and stress response (Figure 6A and Table S8). Predicted *cis*-elements that could respond to hormones such as auxin, gibberellin, salicylic acid, abscisic acid, and MeJA (methyl jasmonate) were identified. Stress-responsive elements such as light, defense, wound, drought, and low temperature were also predicted in *GmNTPs*. It is worth noting that all *GmNTP* members have many light-responsive elements, suggesting that *GmNTP* genes may play important roles in response to environmental factors, especially light stress. Previous studies have shown that HESO1 and URT1 act cooperatively with SKI2 to regulate photosynthesis by RNA surveillance of Calvin cycle genes [38].

The number of coding DNA sequences (CDSs) in *GmNTP* genes ranges from 6 to 15, while the number of exons varies from 6 to 20 (Figure 6B and Table S9). Among them, *GmNTP2* and *GmNTP9* exhibit the simplest gene structures, while *GmNTP3* displays the most complex structure. By analyzing the distribution of exons, it was observed that G1 subfamily genes shared similar gene structures among their homologs. For instance, *GmNTP4*, *GmNTP5*, *GmNTP7*, *GmNTP13*, *GmNTP15*, and *GmNTP16* shared similar gene structure distributions and lengths, indicating that they may originate from a common gene through a series of duplication events and subsequent evolution.

In contrast, only approximately half of the genes in the G2 subfamily exhibit similar gene structures as described above. For example, *GmNTP10* and *GmNTP14*, as a pair of homologenes, have similar gene structures and comparable CDS numbers and lengths to *AtHESO1*. However, *OsNTP3* exhibits notable differences in gene structure compared to other *AtHESO1* homologenes. The differences were similarly found in the branches of *OsNTP10* and *AtURT1*, possibly because *Oryza sativa* is a monocotyledon, whereas *Arabidopsis* and soybean are dicotyledons.

### 2.9. Expression Analysis of GmNTP Genes at Different Tissues and Developmental Stages

**Figure 7:**
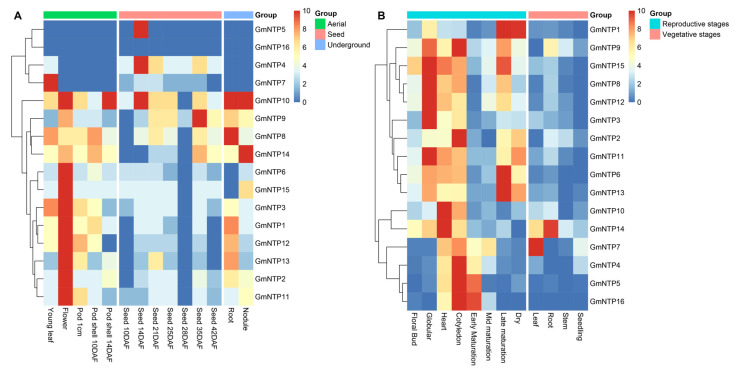
Tissue-specific expression of *GmNTP* genes. (**A**) Expression data for 14 tissues, including root, nodule (underground tissues), leaf, flower, pod-shell 10 days after flowering (DAF), pod-shell 14 DAF, one cm pod (aerial tissues), and different stages of seed development (underground). (**B**) Expression data for *GmNTP* genes at various developmental stages, including root, stem, leaf, flower, seeding (vegetative stages), buds, and different stages of seed development (reproductive stages).

Gene expression data of 14 soybean tissues were downloaded through Soybase, including young leaf, flower, 1 cm pod (7 days after flowering, DAF), pod shell (10 DAF and 14 DAF), seed (10, 14, 21, 25, 28, and 35 DAF), root and node (Figure 7A and Table S10a). All these organizations can be divided into three groups: underground, aerial, and seed. The expression analysis of *GmNTP* genes in different tissues showed that GmNTPs have different tissue-specific expression patterns. Out of the 16 *GmNTP* genes, 10 were expressed in all tissues, including *GmNTP1*, *GmNTP2*, *GmNTP3*, *GmNTP8*, *GmNTP9*, *GmNTP10*, *GmNTP11*, *GmNTP12*, *GmNTP13* and *GmNTP14*. Some genes were specifically highly expressed in flowers compared to other tissues, such as *GmNTP1*, *GmNTP2*, *GmNTP3*, *GmNTP6*, *GmNTP11*, *GmNTP12*, *GmNTP13*, and *GmNTP15*. *GmNTP10* and *GmNTP14* have high levels of expression in all tissues. However, *GmNTP16* was not expressed in these tissues, and *GmNTP5* was only expressed in the seeds at 14 days after flowering, suggesting that they may have special functions. A total of 14 *GmNTP* genes was expressed on the stage of seed maturation, while only GmNTP4, *GmNTP7*, and *GmNTP9* had high levels of expression in the seeds at 28 days after flowering.

Moreover, we analyzed the expression of *GmNTP* genes in the reproductive and vegetative stages using publicly available soybean data (GSE29163). All *GmNTP* genes had higher levels of expression in different phases of the reproductive stage (Figure 7B and Table S10b). It is worth noting that the expression of *GmNTP16* changes with the different phases of seed development (globular, heart, cotyledon, early, mid, late maturation, and dry), suggesting the function of *GmNTP16* in relation to seed development. The examination of 2D hierarchical clustering analysis suggested that the expression patterns of *GmNTP8/9/12/15*, *GmNTP2/3/6/11*, *GmNTP10/14*, and *GmNTP4/5/7/16* were similar, but that *GmNTP1* was different from the others. The expression level of *GmNTP1* is increased in the process of seed maturation, suggesting that its functions are related to nutrient storage.

### 2.10. Expression Analysis of GmNTP Genes under Abiotic Stress

**Figure 8:**
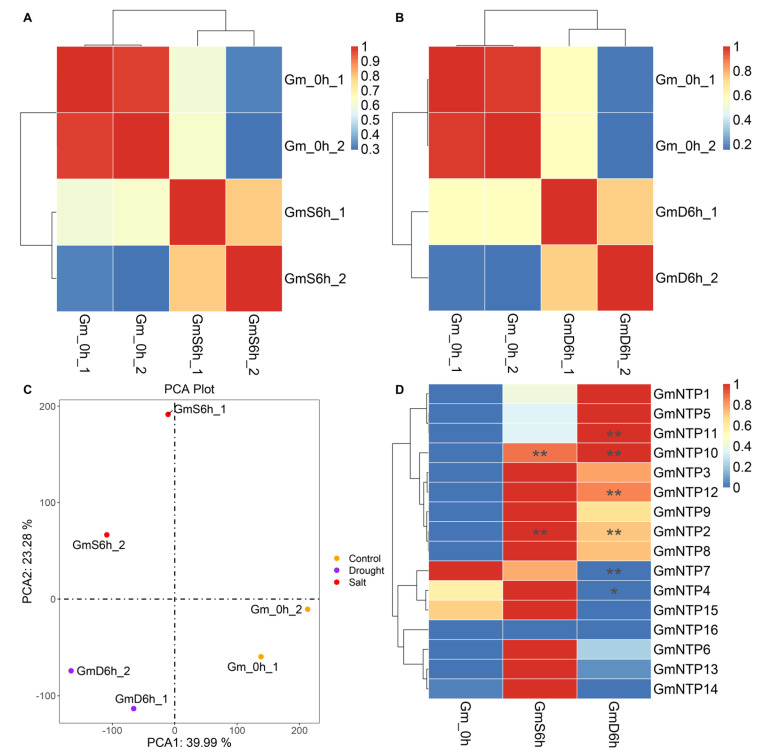
Sample correlation and principal component analysis (PCA) analysis of all transcripts and expression analysis of *GmNTP* genes under abiotic stresses. (**A**,**B**) Sample correlation analysis of the control and experimental groups. Gm_0h, wild-type soybeans; GmS6h, soybeans under salt stress for 6 h; GmD6h, soybeans under drought stress for 6 h. (**C**) PCA plot of transcriptome results. (**D**) Expression heatmap of *GmNTP* genes under salt or drought stress. The different symbols represent the level of significant difference, *, *p* < 0.05; **, *p* < 0.01.

To explore the expression patterns of *GmNTP* genes under abiotic stress, 4-week-old soybean seedlings were subjected to high-salt and drought stresses for 0 h (control group) and 6 h (experimental group). Total RNA was extracted, and libraries were constructed for RNA-seq. Principal component analysis (PCA) and sample correlation analysis were conducted between control and experimental samples (Figure 8 and Table S11). Hierarchical clustering revealed that the same treatment had a high similarity in two repeats, but there was a significant difference between the control and experimental groups.

The analysis found that 6026 genes were upregulated and 6059 genes were downregulated under salt stress; 7107 genes were upregulated and 8438 genes were downregulated under drought stress. Meanwhile, 4135 genes were similarly upregulated under salt or drought stress, but 4350 genes were similarly downregulated (Figures S5 and S6). Interestingly, most *GmNTP* genes were upregulated after salt or drought treatments, including *GmNTP1*, *GmNTP2*, *GmNTP3*, *GmNTP5, GmNTP6*, *GmNTP8*, *GmNTP9*, *GmNTP10*, *GmNTP11*, *GmNTP12*, and *GmNTP13* (Figure 8D). Among them, *GmNTP2* and *GmNTP10* were significantly increased, by 2.88-fold and 2.2-fold, under salt stress, respectively. The expression of *GmNTP4*, *GmNTP14*, and *GmNTP15* was induced by salt stress but was inhibited by drought stress. *GmNTP7* was similarly downregulated under salt or drought stress, and significantly decreased by 13-fold under drought stress. GmNTP4 also was significantly decreased 3.03-fold under drought stress. Moreover, *GmNTP2/10*/*11/12* were significantly increased 3.03-fold, 3.21-fold, 3.11-fold, and 2.21-fold under drought stress, respectively. The expression of *GmNTP16* was not detected under salt or drought stress.

**Figure 9:**
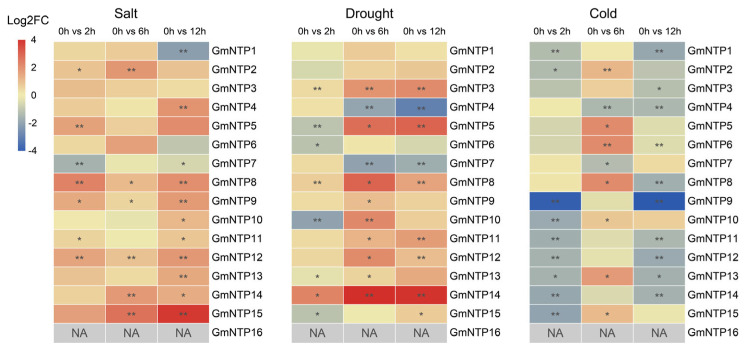
The differential expression analysis of GmNTP genes under salt, drought, and low-temperature stresses. Heat map illustrating the changes in expression of GmNTP genes identified by qRT-PCR under abiotic stress. NA means did not detected the expression by qRT-PCR. The data are shown as the mean ± SD of three independent biological replicates. The different symbols represent the level of significant difference, *, *p* < 0.05; **, *p* < 0.01.

To verify the transcriptome data, we detected *GmNTP* gene expression after 2, 6, and 12 h under high-salt and drought stress conditions by qRT-PCR; the results were consistent with the RNA-seq results (Figure 9 and Figure S7). Most *GmNTP* genes, except *GmNTP16,* were significantly upregulated under salt stress. *GmNTP1/2/6/14* showed the highest expression levels after 6 h of salt treatment, which were 1.6-fold, 3.48-fold, 3.06-fold, and 3.35-fold, respectively. *GmNTP4/5/9/10/11/12/13/15* showed the highest expression levels at 12 h of salt treatment, which were 3.64-fold, 4.07-fold, 3.28-fold, 2.14-fold, 1.73-fold, 3.41-fold, 2.69-fold, and 13.75-fold, respectively. *GmNTP7* was significantly decreased 1.4-fold to 2.9-fold under salt stress. However, GmNTPs have increasing or decreasing expression under drought stress. *GmNTP3/5/11/13/15* increased with drought treatment time, with significant increases of 4.15-fold, 7.32-fold, 3.18-fold, 1.46-fold, and 1.6-fold. GmNTP4/7 decreased with drought treatment time, with significantly decreases of 7.35-fold and 4-fold. The highest expression levels of most GmNTP genes occurred after 6 h of cold treatment, but significant downregulation was observed with 2 h or 12 h of treatment. For example, GmNTP2/10/13/15 were significantly increased by 2.2-fold, 1.6-fold, 3.3-fold, and 2.08-fold after 6 h of treatment, but were significantly decreased by 2.5-fold, 3.44-fold, 3.03-fold, and 3.92-fold after 2 h of cold treatment. *GmNTP4/7* were similarly significantly decreased by 2.7-fold and 2.43-fold after 6 h of cold treatment, showing a different expression pattern with other *NTP* genes. *GmNTP16* was not detected in any of the samples during the three stress treatments, which may be because it is only expressed in the reproductive stage.

### 2.11. Subcellular Localization of GmNTP2 and GmNTP14

**Figure 10:**
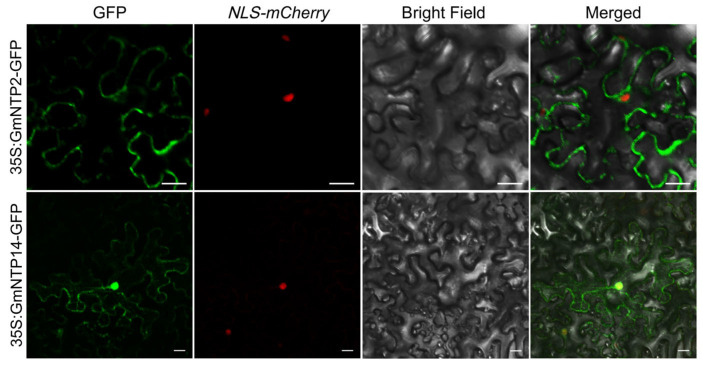
Analysis of the subcellular localization of GmNTP2 and GmNTP14. GmNTP2-GFP and GmNTP14-GFP colocalized with NLS-mCherry, which is a marker of nuclear localization. For each construct, 10–20 cells were analyzed, and a common nuclear location pattern was observed. However, GFP fluorescence was also observed in the cytoplasm. Scale bar, 20 μm.

In *Arabidopsis*, NTP proteins are mainly located in the nucleus and cytoplasm and play a role in nucleotide transfer. To determine whether the localization of NTP proteins in soybean was consistent with their homologenes, *GmNTP2* and *GmNTP14* were selected for subcellular localization. *GmNTP2* is homologous to *AtURT1*, and *GmNTP14* is homologous to *AtHESO1*. *GmNTP2* and *GmNTP14* were fused to the N-end of GFP and coexpressed in tobacco (*N. tabacum*) leaves with NLS-mCherry. The results showed that the green fluorescence signal (GFP) overlapped with the red fluorescence signal (mCherry), suggesting that GmNTP2 and GmNTP14 were located in the nucleus (Figure 10). Furthermore, the GFP signal also exists in the cytoplasm and is the same as that of *AtHESO1* and *AtURT1* [29].

## 3. Discussion

Soybean is one of the most important oilseed crops globally; however, its yield is susceptible to various abiotic stresses [39]. Global climate change introduces challenges like soil salinization and drought, adding constraints to soybean production. Meanwhile, as a warm-season plant, soybean is highly sensitive to low-temperature stress. The miRNAs in soybeans play a crucial role in stress resistance [40,41]. Soybean’s miR482bd-5p is associated with salt and osmotic stress by regulating its potential HEC1 and BAK1 targets. However, the *MIR482bd* gene repressed histone deacetylases (HDACs) under salt stress [42]. The overexpression of gma-miR172c in *Arabidopsis* resulted in enhanced tolerance to water deficit and salt stress while increasing ABA sensitivity, influencing flowering time by modulating the expression of related genes [43]. Moreover, research has revealed that gma-miR172c influences the tolerance of soybean roots to stress by affecting the target gene NNC1 (Nodule Number Control 1) [44]. Many small RNAs have been experimentally confirmed to play a role in plant responses to abiotic stresses [5,45,46,47,48]. NTP, as an enzyme that has the function of terminal modification, plays an important role in the process of sRNA biogenesis and degradation [8,23]. For example, miR165/166 is recognized by SDN1 and SDN2 in *Arabidopsis*, promoting its 3’ truncation, uridylation, and degradation [49]. Simultaneously, miR166, as a highly conserved miRNA family among land plants, has an important role in various developmental processes and responses to biotic and abiotic stress in major crop plants [50,51]. In soybean, it is possible that plants regulate miRNA expression through NTP in response to biotic and abiotic stresses. Genome-wide identification of *NTP* genes has been studied in several plants [1,23,24]. Therefore, we conducted a comprehensive bioinformatics analysis of the GmNTP family and identified certain *GmNTPs* that respond to abiotic stress, providing potential avenues for exploring the functionality of the GmNTP family.

In this study, 16 *GmNTP* genes were identified in the soybean genome. Previous studies have shown that there are 10, 13, and 24 NTP genes in *Arabidopsis*, rice, and maize, respectively [1,23,24]. The characteristics of NTPs are similar to those in other species, including pI and protein length (Table 1). However, differences in characteristics are suggestive of their functional diversity, especially considering the importance of pIs to protein function. Several investigations show that nuclear proteins have a pI of 4.5 to 10.0 and cytoplasmic proteins have a pI of 5.0 to 6.0 in eukaryotes [52]. The pIs of GmNTPs have an average of 6.70, ranging from 5.3 to 9.68. Small RNA biosynthesis and processing sites are mainly found in the nucleus and cytoplasm, and HESO1 and URT1 were simultaneously located in the nucleus and the cytoplasm [29]. Most of the genes coexpressed with *GmNTP* were predicted to be localized in the nucleus and the cytoplasm (Figure S4). At the same time, GmNTP2 and GmNTP14 had the same subcellular location as their homologous genes HESO1 and URT1 (Figure 10). The results revealed that *GmNTP2* and *GmNTP14* have possibly an important function, similar to *HESO1* and *URT1*.

The phylogenetic tree divides NTPs into two clades and show the highly evolutionary relationship (Figure 1). The number of *GmNTP* genes is almost double that of *Arabidopsis* and the phylogenetic tree has almost more than two *GmNTP* genes in the same clade. The NTP family contains 15 duplicated gene pairs and 7 collinear gene pairs, implying there is likely to be functional redundancy between *GmNTP* genes (Figure 2A,B). This redundancy poses challenges for studying *NTP* genes, making it difficult to investigate their functions through single-gene knockout [12,26]. Meanwhile, the expansion of *NTPs* may be the results of segmental duplication and whole-genome duplication [53]. The number of collinear gene pairs was obviously different between *Arabidopsis* and rice. This may be related to the fact that both soybean and *Arabidopsis* belong to dicotyledons. The ka/ks value is widely used to represent an indicator of selective pressure [54]. These gene pairs belong to purifying selection in evolution, manifesting the evolution of NTP gene with strong constraints, which may contribute to their functional stability. This evolutionary relationship is also shown in their gene structure because the exon distribution of NTPs in the same clade is basically similar (Figure 6B and Table S9). Similar evidence was also found in protein interactions and gene coexpression network (Figure 5, Figures S3 and S4). GmNTP2 and GmNTP9 simultaneously interacts with GmNTP3/8/12. The homologous genes have coexpression relationship.

It is worth noting that the core domain of NTPs is closer to the N-terminus compared to PAP_assoc and corresponds to Motif 4 (GS--D-D) and Motif 10 (A-VP--D) (Figure 3A and Figure S1). The conserved GS and D are retained across all NTP genes and present mono-helices and sheets in their predicted tertiary structure (Figure 4B and Figure S2). Motif 10 has a conserved A-VP--D structure and only exists in the G2 subfamily. Zhao et al. constructed the proteins of HESO1, replacing DXD to AXA in this site, but could not show uridylation function [54]. Zhu et al. compared the crystal structures of URT1 containing a D547A mutation and its complex with UTP (URT1-UTP) and found their structure was almost identical [33]. The D547A mutation lacks uridylation function, but R531A and N552A fail to add the function of the long U-tail, which may be important for extension of the U-tail at the 3′ end of miRNAs. Indeed, the three aspartic acids forming a catalytic aspartate triad become the core of the active site of the enzyme, which has been found in REF1 [34,35,36,55]. This phenomenon is also found in the plant *NTP* genes, but NTPs with the function of uridylation and their homologous genes have a conserved SCD structure in three aspartic acids, which may be an important clue to the specific addition of uridylate (Figure 4B).

In terms of tissue-specific expression, *GmNTP* genes are expressed in the leaf, root, nodule, flower, pod, and seed, but not much in the stem, which may be associated with the nucleotide transferase activity and division of work (Figure 7A). Furthermore, GmNTP10/14 exhibit relatively high expression in root nodules. Research suggests that the early whole-genome duplication events in legumes may provide a rich genetic basis for the stable and effective nitrogen-fixing nodules [56]. The miR172 in soybeans influences the formation of root nodules [57]. Therefore, we speculate that the occurrence of replication events of HESO1 and URT1 genes in soybeans may be related to the regulation of miRNA processing or degradation in soybean nodules, thus impacting nitrogen fixation in soybeans. *GmNTP5* and *GmNTP16* are clearly different from the other genes and are a pair of genes of the same clade. *GmNTP5* is expressed almost exclusively in the early stage of seed development, but *GmNTP16* is not detected in any tissues except reproductive tissue, which may be the reason why the coexpressed gene has not been detected. (Figure 7B and Figure S4).

RNA-seq and qRT-PCR analysis suggest that the expression of NTP genes was significantly affected by stress (Figure 8 and Figure 9). Meanwhile, most of the GmNTP genes contain plant hormone response elements and stress response elements (Figure 6A). Among them, ABA and MeJA response elements are the most common, indicating that GmNTPs may be involved in ABA- and JA-dependent signaling pathways. It is worth noting that 133 light response elements were detected in the promoter of GmNTPs, and most of the GmNTPs contained G-box, GT1, TCCC, and TCT motifs. Indeed, the response of NTP to different abiotic stresses and hormones was also found in other plants, such as rice and maize [1,24]. However, this study primarily focuses on the effects of yield-related abiotic stress on the NTP family, an important regulator of the stability of small RNAs. Under salt treatment, *GmNTP2* was significantly elevated, by 3.69-fold, and *GmNTP5* was elevated by 7.5-fold, which may be attributed to TC-rich repeats. *GmNTP4* and *GmNTP10* were significantly changed under drought treatment, and *GmNTP5* was upregulated 20-fold, which may be due to MBS elements. *GmNTP14*, as the homolog of *GmNTP10* and *AtHESO1*, was upregulated under salt stress but downregulated under drought treatment. *GmNTP2* and *GmNTP9*, as homologs of *AtURT1*, were upregulated under salt or drought stress.

In addition, cold stress seriously affects the yield of temperate crops, but also affects the expression of NTP family genes. Most *GmNTP* genes are highly expression at 6 h under cold stress, but significantly downregulated at 2 h and 12 h. Some conserved gma-miRNAs have been shown to regulate plant salt, drought, and cold responses [58,59,60]. For example, miR166, miR171, and miR169 were shown to regulate plant cold response in soybean [60]. When the 3’ end is unmethylated, these small RNAs were nontemplatetrically added uridylate by HESO1 and degraded [8]. miR166 and miR169 were also found to regulate drought response [58]. gma-miR169c exerts a negative regulatory role in response to drought stress by inhibiting its target gene, nuclear factor Y-A (NF-YA), although how gma-miR169c functions in response to drought stress remains unclear [61]. It is possible that NTP to response drought stress by regulating the level of gma-miR169. In addition, NTPs have another role in mRNA tailing, which suggests that GmNTPs may regulate the expression of stress-related genes by directly modifying their transcripts [28].

NTPs are crucial proteases that regulate small RNAs at the post-transcriptional level. Our results showed that the NTP family is a conserved in plants and plays a role in the stress response. However, the regulatory mechanism of NTPs requires further study. The results provide novel insight into the regulatory networks underlying the response of small RNAs to abiotic stress. Our search has important implications for further investigations into the functions of NTP family genes in soybeans.

## 4. Materials and Methods

### 4.1. Identification and Phylogenetic Tree Construction of GmNTPs

The genome sequences, coding sequences (CDS) and protein sequences of soybean (*Glycine max* ‘Williams 82′) were obtained from the soybean genome database (https://www.soybase.org/, accessed on 5 May 2023) and the Phytozome database (https://phytozome-next.jgi.doe.gov/, accessed on 5 May 2023). The HMM information of the NTP_transf_2 domain (PF01909) was obtained from the Pfam database (https://www.ebi.ac.uk/interpro, accessed on 9 May 2023), and the candidate members containing this domain were screened using HMMER3.0 program (http://hmmer.janelia.org/, accessed on 9 May 2023). The domain information of the candidate protein was analyzed and searched for proteins containing the NTP_transf_2 domain. The identified GmNTP protein information was obtained from the UniProt database (https://www.uniprot.org, accessed on 9 May 2023), such as amino acid length, molecular weight, isoelectric point, and chromosome position.

The NTP family member sequences for *Arabidopsis thaliana* and *Oryza sativa* were obtained from the UniProt database (Table S2). A total of 39 NTP proteins from *Arabidopsis thaliana* (10 NTPs), rice (13 NTPs), and soybean (16 NTPs) were multiple-sequence aligned using the ClustalW tool provided by MEGA7 (v7.0.21). Subsequently, the phylogenetic tree of soybean, rice, and *Arabidopsis* was constructed using MEGA7 (v7.0.21) by the neighbor-joining (NJ) method with the bootstrap parameter set to 1000 [62]. The phylogenetic tree was visualized using Evolview (https://www.evolgenius.info/evolview, accessed on 12 May 2023) [63].

### 4.2. Chromosomal Location and Collinearity Analysis

The distribution and physical locations of GmNTP family protein-coding genes on soybean chromosomes were obtained from the Phytozome database. Multiple sequence alignment was performed using the ClustalOmega (https://www.ebi.ac.uk/tools/MSA/clustalo/, accessed on 12 June 2023) to record sequence similarity among *GmNTP* genes [64]. The location of *GmNTP* genes on soybean chromosomes was marked using MG2C (http://mg2c.iask.in/mg2c_v2.1/, accessed on 12 June 2023) [65]. The ratio of similarity was more than 70% and the alignment sequence length was more than 80% of the longest gene that was identified as a gene duplicated event. The duplicated gene pairs were linked to the map with a straight line. MCScanX was used to assess the duplication events and to analyze the collinearity between species using the default parameters [66]. The data were further analyzed using Advanced Circos v2.0 for collinearity within soybean species and Multiple Synteny Plot v1 tool for collinearity analysis among soybean, rice and *Arabidopsis* genes [67]. The Ka/Ks ratios in *GmNTP* genes were computed using KaKs Calculator 3.0 [68].

### 4.3. Conserved Motifs, Secondary Structure, and Conserved Domains Analysis of GmNTPs

The conserved motifs of NTPs of *Arabidopsis thaliana*, rice, and soybean were generated by MEME (https://meme-suite.org/meme/tools/meme, accessed on 22 June 2023) [69]. The secondary structures were predicted by the SOPMA tool (https://npsa-prabi.ibcp.fr/cgi-bin/npsa_automat.pl?page=npsa_sopma.html, accessed on 24 June 2023) [70]. The output results included the proportions of various secondary structures, such as alpha helix, beta turn, and random coil. The conserved domains were analyzed by the HMMER3.0 program. The results of conserved motifs, secondary structure, and conserved domains were visualized by TBtools (v2.012) [67].

### 4.4. Gene Structure and Cis-Acting Elements Analysis of GmNTPs

The exon/intron locations of NTPs of *Arabidopsis thaliana*, rice, and soybean were drawn by TBtools (v2.012) [67]. The cis-acting elements of 1500 bp upstream sequences from the translation start codon (ATG) of all *NTP* genes were predicted by the PlantCARE website (http://bioinformatics.psb.ugent.be/webtools/plantcare/html/, accessed on 18 June 2023) and retained phytohormone and stress response elements [71]. The results were visualized by TBtools (v2.012) [67].

### 4.5. Tertiary Structure Prediction of GmNTP Proteins

The tertiary structure prediction of GmNTP proteins was performed using AlphaFold2 (v2.3.2) [72]. The highest confidence score (pLDDT) among the five obtained models was selected as the final result. To understand the position of the conserved structure, multiple sequence alignment was conducted by MUSCLE (v3.8.1551) and visualized using JalView (v2.11.3.0) [73,74]. Finally, the territorial structure visualization of GmNTP proteins was performed using PyMOL (v2.5.7) [75].

### 4.6. Protein Interaction Network and Gene Co-Expression Analysis of GmNTP

Protein interaction network of single GmNTP and all GmNTPs was predicted using STRING v.12 (confidence limit is 0.4) [76]. Meanwhile, the GO enrichment results generated by STRING were visualized using the ggplot2 package in R. We constructed a co-expression network for the *GmNTP* gene using ATTED-Ⅱ ver.11.1 and visualized the results using Cytoscape software (v3.10.1) (Coex option: add many genes; PPI option: do not draw PPI) [77,78].

### 4.7. Expression Profiles of GmNTP Genes at Different Tissues and Developmental Stages

The normalized RNA-seq data for *GmNTP* genes in 14 tissues was downloaded from Soybase (http://soybase.org/soyseq/, accessed on 5 May 2023) including three groups, such as underground tissues: root, nodule; aerial tissues: young leaf, flower, one-cm pod, pod-shell 10 DAF, pod-shell 14 DAF; seed development stages (seed of 10 DAF, 14 DAF, 21 DAF, 25 DAF, 28 DAF, 35 DAF, and 42 DAF). The RNA-seq data (GSE29163) of *G. max*, describing the expression of *GmNTP* genes in two different developmental stages, including 12 soybean tissues, was published by Goldberg RB et al. [79]. After normalization, the values were used for clustering and visualized using the R software (v4.3.1) package pheatmap (v1.0.12).

### 4.8. Plant Materials and Stress Treatments

Healthy plump soybean seeds (*Glycine max* (L.) cultivars Williams 82) were selected and subjected to overnight vernalization at 4 °C. To enhance permeability, the seed coat was gently scratched after disinfection and cultured under suitable conditions before transplanting into the culture solution. The culture system was sealed with plastic wrap and the plastic wrap was removed after the seeds have germinated. Germinated soybean seedlings were cultured with a quarter concentration of Hoagland nutrient solution at a temperature of 24 °C, with a photosynthetic effective radiation of 250 μmol m^−2^ s^−1^ under 12 h day/12 h night. After 4 weeks of cultivation, soybean seedlings developed 4 true leaves, and seedlings at the same developmental stage were subjected to three abiotic stress treatments. Salt treatment was initiated by nutrient solution containing 300 mM NaCl. Drought treatment was initiated by moving the plants to 3 mm filter paper. Cold treatment was initiated at 4 °C. Each treatment was applied for four different time periods: 0, 2, 6, and 12 h, and involved at least three biological replicates. The seedlings were harvested with liquid nitrogen and placed in the refrigerator at −80 °C for subsequent experiments.

### 4.9. Transcriptome Sequencing, Data Processing, and Gene Expression Analysis

We cultivated soybean seedlings for 4 weeks and subjected them to salt or drought stress for 6 h. Total RNA from 6 samples, including two control samples (0 h), two salt stress samples (6 h), and two drought stress samples, were sent to Genergy Biotechnology (Shanghai, China) for sequencing on an Illumina^®^ Hiseq3000 (San Diego, CA, USA). Every treated sample contained three seedlings. The raw data were processed to remove the adapter sequences and low-quality reads using Skewer software (v0.2.2) (Table S11) [80]. The clean reads were mapped to the reference genome sequence of soybean (Wm82.a4.v1) using STAR (v2.5.3a) software [81]. Different expression genes (DEGs) were analyzed using DESeq2 (v1.16.1) [82]. Genes with a false discovery rate (FDR) of < 0.05 and |log2 (fold change)| ≥ 1 detected by DEseq2 were classified as differentially expressed. *GmNTP* gene family members identified in the data were analyzed (Figure S6).

### 4.10. RNA Extraction and qRT-PCR Analysis

Total RNA from the stored plants was extracted with Trizol and cDNA was obtained using the BeyoRT™ II cDNA kit (Beyotime, Shanghai, China). The quantitative analysis was performed by real-time PCR in conjunction with Universal SYBR qPCR Mix (Biosharp, Hefei, Anhui, China) on a LightCycler 480 System (Roche, Vienna, Austria) in accordance with the manufacturer’s instructions. The primers used are listed in Table S1. The following reaction conditions were applied: pre-degeneration at 95 °C for 30 s, followed by 40 cycles of denaturation at 95 °C for 15 s and 60 °C for 30 s, and the melting curve was evaluated form 60 °C to 95 °C. The relative transcript levels of the *GmNTP* genes were calculated according to the 2^−ΔΔCT^ method [83]. The melting peaks of the candidate genes are displayed in Figure S8.

### 4.11. Construction of Vectors and Subcellular Localization

The subcellular localization of *GmNTP* genes was predicted using Plant-PLoc (http://www.csbio.sjtu.edu.cn/bioinf/plant, accessed on 5 June 2023) by uploading protein sequences [84]. The coding sequence of *GmNTP2* and *GmNTP14* lacking the stop codon was amplified and were linked to the 5′ end of the mGFP of pGreenII-35S-mGFP, and then transformed into DH5α. The resulting fusion expression vectors, pGreenII-GmNTP2 and pGreenII-GmNTP14, were validated by sequencing. Subsequently, the validated fusion expression vectors were transformed into *Agrobacterium strain* GV3101 (pSoup). The nuclear marker was created by linking the NLS-sequence to pGreenII-35S-mCherry and was validated with infected *Nicotiana* leaf cells. The primer sequence information can be found in Table S1. According previous methods, the fusion expression vector was transformed into *Agrobacterium*, and then infected with *Nicotiana benthamiana* [85]. The infected leaf epidermal cells were imaged using a confocal laser microscope (OLYMPUS FV3000, Tokyo, Japan) at an excitation wavelength of 488 nm to detect GFP and at an excitation wavelength of 561 nm to detect mCherry.

### 4.12. Statistical Analysis

In this study, the qRT-PCR results were reported as the mean of three independent experiments and each experiment included three technical replicates. The significant differences between treatments were analyzed using standard deviation and one-way analysis of variance (ANOVA). The significant differences between the control group and the treatment groups were assessed by Student’s *t*-test. The value of *p* < 0.05 was used as the significance threshold (* *p* < 0.05 and ** *p* < 0.01).

## 5. Conclusions

To summarize, we have identified 16 members of the NTP family in soybeans. Evolutionary analysis and gene co-linearity analysis across multiple species revealed a relatively conserved evolutionary pattern in the NTP family genes. The conserved motif and tertiary structure analysis suggests that the catalytic activity of the NTPs was responsible for three conserved aspartic acids. From the analysis, it was observed that some NTP genes exhibit tissue-specific expression, which may be related to their specific functions. RNA-seq and qRT-PCR analysis confirmed the association of soybean NTP genes with abiotic stress responses. Subcellular localization experiments revealed that the homologous genes *GmNTP2* and *GmNTP14* are localized in the nucleus. Our search has important implications for further investigations into the functions of genes in the NTP family in soybean.

## Figures and Tables

**Table 1:** Basic information of the GmNTP gene family in soybean.

Gene Name	Gene ID	Chromosome	Domain	Other Domain	Length (aa)	Mass (Da)	pI	Putative Localization
*GmNTP1*	*Glyma.02G235800*	2	NTP_transf_2	Unknown	782	87,145	6.03	Nucleus
*GmNTP2*	*Glyma.03G020100*	3	NTP_transf_2	PAP_assoc	731	81,750	7.1	Chloroplast
*GmNTP3*	*Glyma.03G032900*	3	NTP_transf_2	Unknown	1547	170,960	6.72	Nucleus
*GmNTP4*	*Glyma.04G189100*	4	NTP_transf_2	Unknown	692	78,386	5.3	Chloroplast
*GmNTP5*	*Glyma.06G144300*	6	NTP_transf_2	Unknown	721	82,370	5.81	Chloroplast
*GmNTP6*	*Glyma.06G158600*	6	NTP_transf_2	Unknown	376	43,316	8.93	Chloroplast
*GmNTP7*	*Glyma.06G176600*	6	NTP_transf_2	Unknown	682	77,424	5.54	Cytoplasm
*GmNTP8*	*Glyma.07G056700*	7	NTP_transf_2	Unknown	1334	148,607	5.82	Nucleus
*GmNTP9*	*Glyma.07G082200*	7	NTP_transf_2	PAP_assoc	732	81,792	7.74	Chloroplast
*GmNTP10*	*Glyma.11G222700*	11	NTP_transf_2	Unknown	455	51,096	9.65	Chloroplast
*GmNTP11*	*Glyma.14G203800*	14	NTP_transf_2	Unknown	780	86,580	6.17	Nucleus
*GmNTP12*	*Glyma.16G025800*	16	NTP_transf_2	Unknown	1335	148,724	5.81	Nucleus
*GmNTP13*	*Glyma.16G055700*	16	NTP_transf_2	Unknown	684	77,377	5.58	Chloroplast
*GmNTP14*	*Glyma.18G034900*	18	NTP_transf_2	Unknown	415	46,676	9.68	Chloroplast
*GmNTP15*	*Glyma.19G092300*	19	NTP_transf_2	Unknown	660	75,146	5.94	Chloroplast
*GmNTP16*	*Glyma.19G092500*	19	NTP_transf_2	Unknown	626	71,256	5.38	Cytoplasm

## Data Availability

Data are contained within the article and Supplementary Materials.

## Supplementary Materials

The following supporting information can be downloaded at: https://www.mdpi.com/article/10.3390/ijms25021115/s1.
