# Supplementary material for: Identification and Expression Analysis of the Nucleotidyl Transferase Protein (NTP) Family in Soybean (Glycine max) under Various Abiotic Stresses
Source: Int J Mol Sci. 2024 Jan 17;25(2):1115. doi: 10.3390/ijms25021115 (PMC10816777; doi:10.3390/ijms25021115)
Supplement: Supplementary file 1 [file ijms-25-01115-s001.zip › Supplement Figures.pdf]

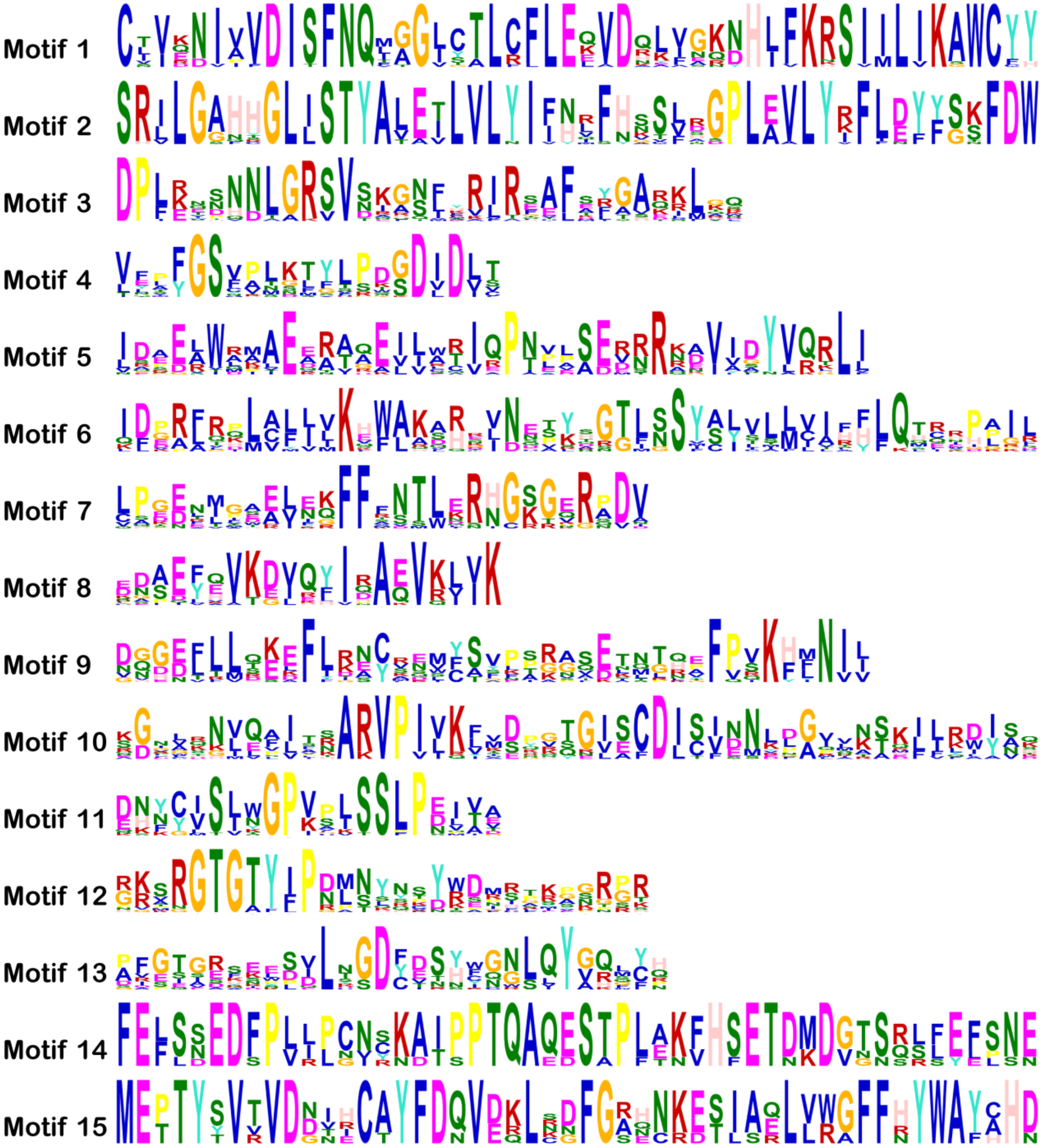

Figure S1. The amino acid composition of conserved motif. A total of 15 conserved motifs were generated in GmNTP protein sequence by MEME.

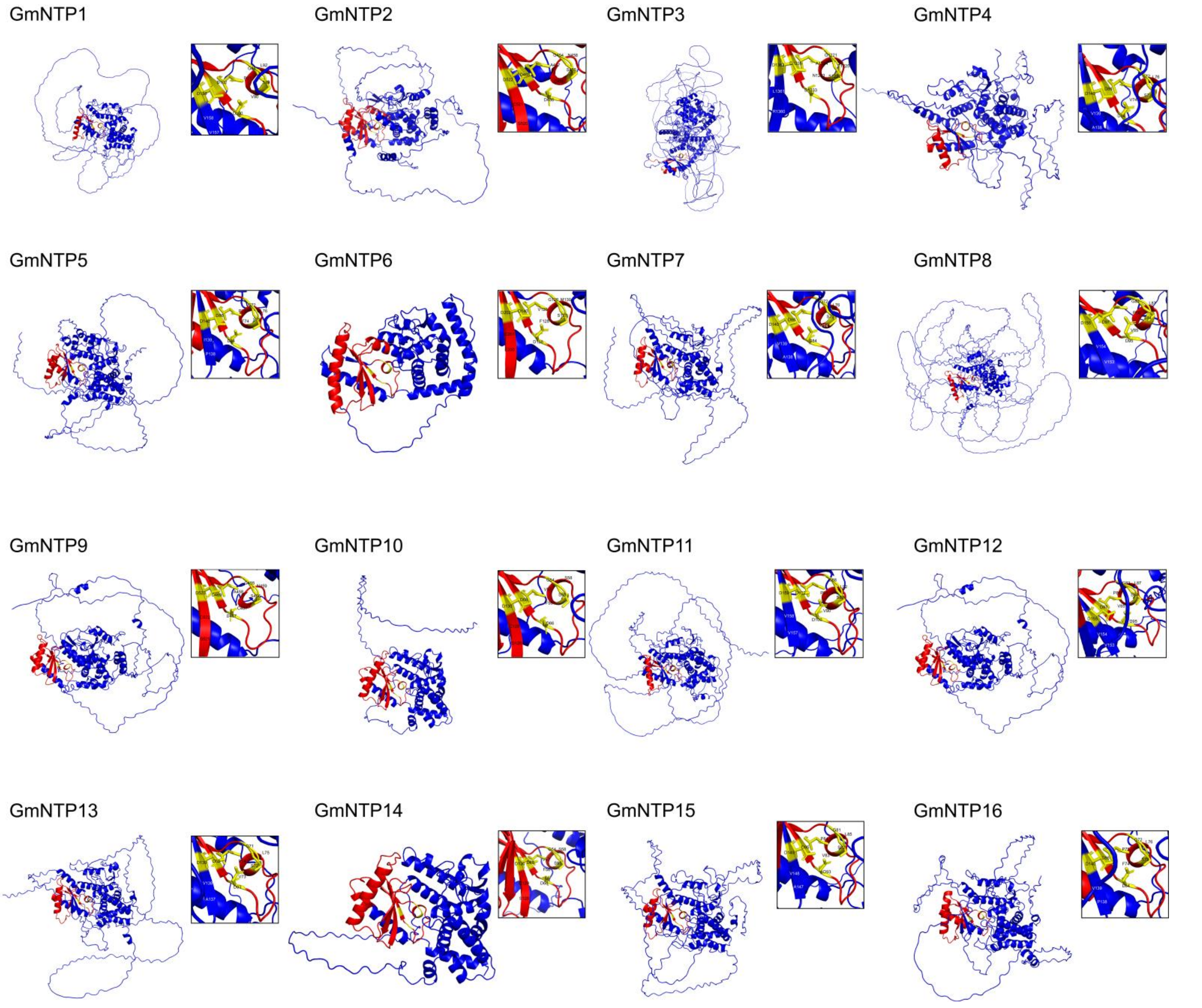

Figure S2. Tertiary structure prediction and analysis of GmNTPs protein. The tertiary structure prediction of GmNTP proteins was performed using AlphaFold2 software. The highest confidence score (pLDDT) among the five obtained models were selected as the final result. The possible active centers formed by three conserved aspartates are shown in the detailed view.

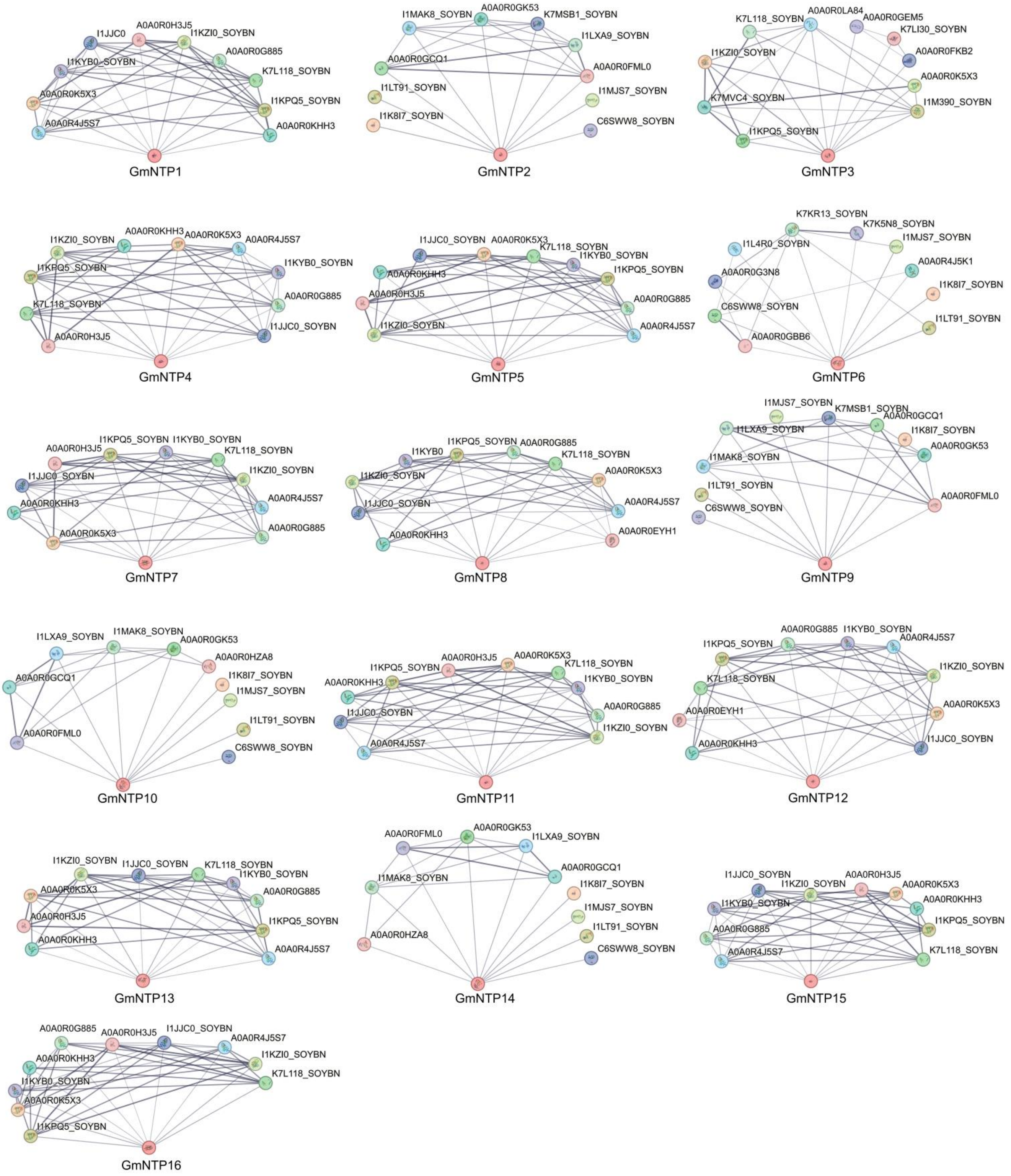

Figure S3. The interaction network of GmNTP with other proteins, where the IDs represent the Uniprot IDs of the proteins. The color of the lines between interacting proteins indicates the strength of their potential interaction, with darker shades suggesting a higher likelihood. The deep red circle represent the GmNTP proteins in the network.

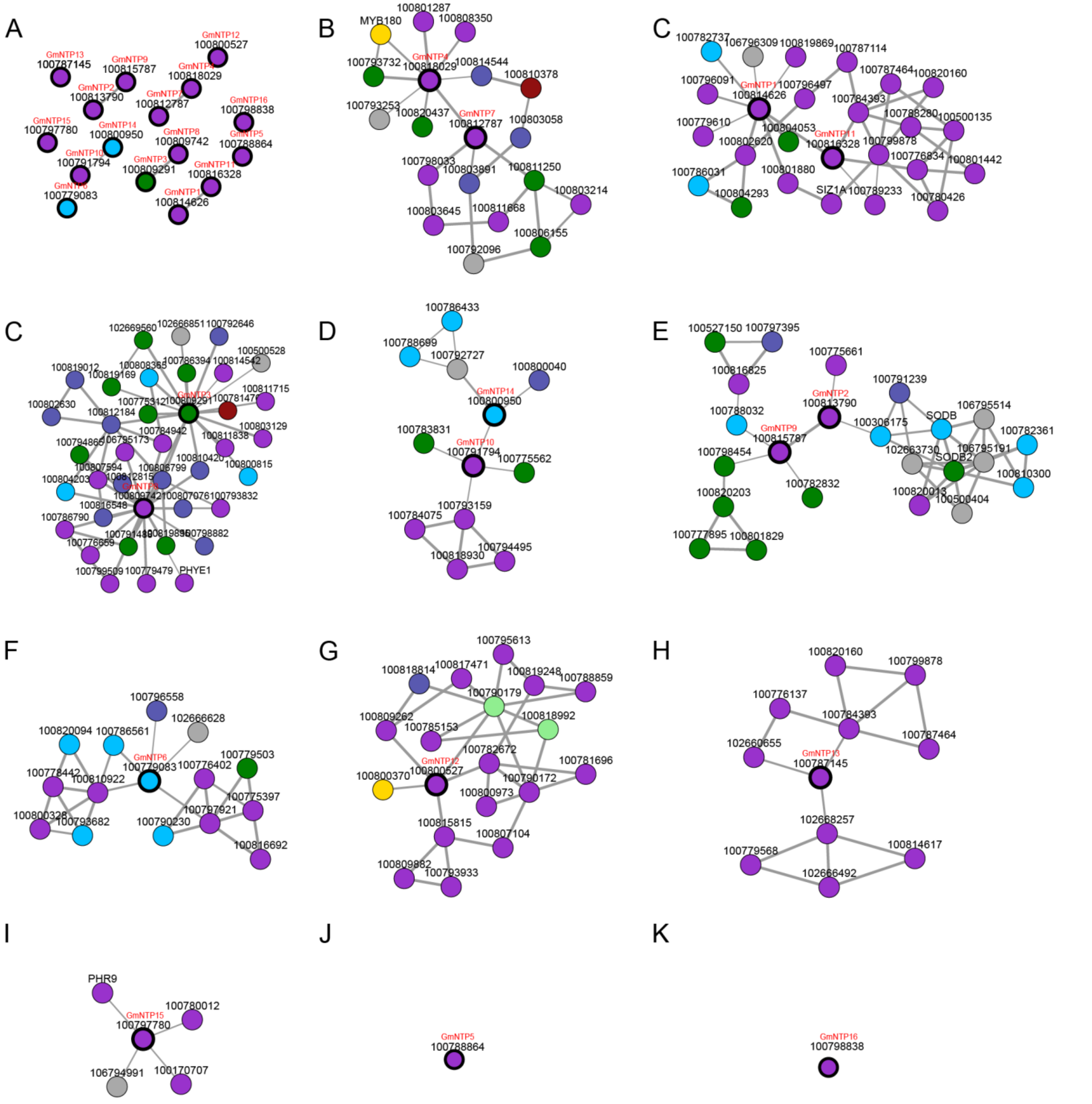

Figure S4. The co-expression networks of GmNTP family genes in soybean. (A) Five pairs co-expression genes were found in GmNTP family. (B-K) The clustered co-expression nodes of *NTP* genes extends out of the subnetwork. The id above each node represents their LOC number in NCBI. The color inside the circle represent their possible subcellular localization. The purple represents nucleus, the yellow represents mitochondria, the green represents chloroplast, the light blue represents cytoplasm, the dark blue represents plasma membrane, the light green represents Golgi apparatus, the brown represent vacuole, the grey represents unclear.

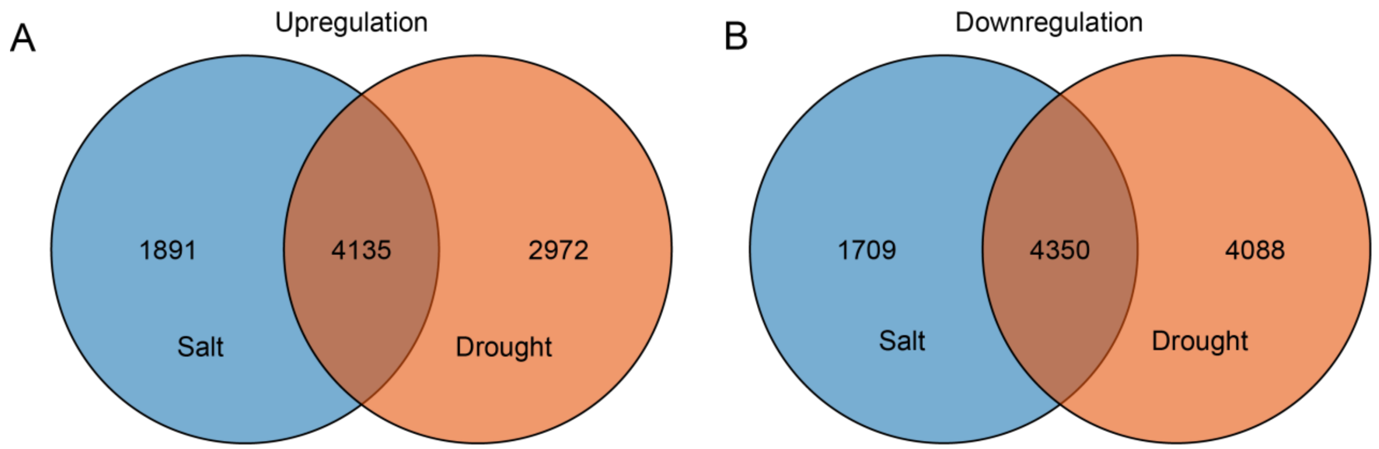

Figure S5. Venn diagram of the number of different expression genes (DEGs) in soybean under two treatment conditions. (A) The number of genes that are upregulated under salt or drought stress. (B) The number of genes that are downregulated under salt or drought stress.

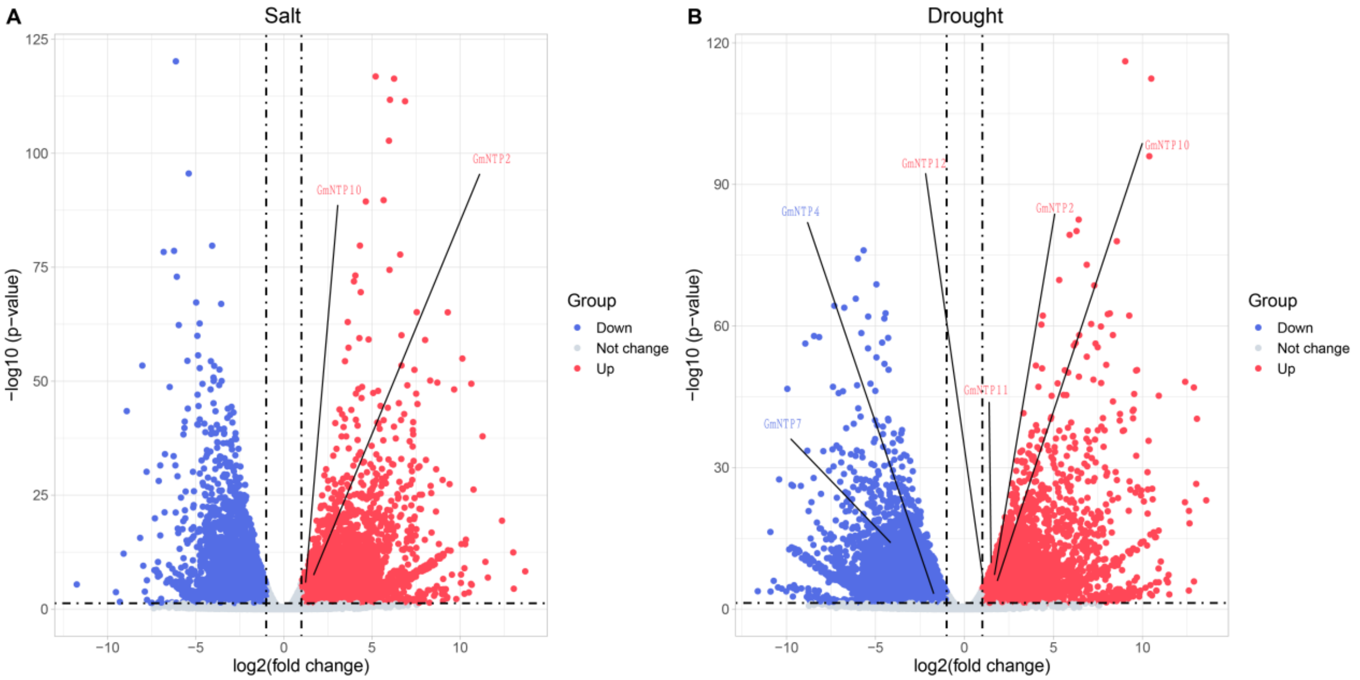

Figure S6. Volcano plot of differential gene expression under two treatment conditions. (A) Volcano plot of DEGs in soybean under salt stress. (B) Volcano plot of GEGs in soybean under drought stress. Blue dots represent genes that are downregulated. Red dots represent the genes that are upregulated. The horizontal axis represents the difference multiple, while the vertical axis represents the significance level.

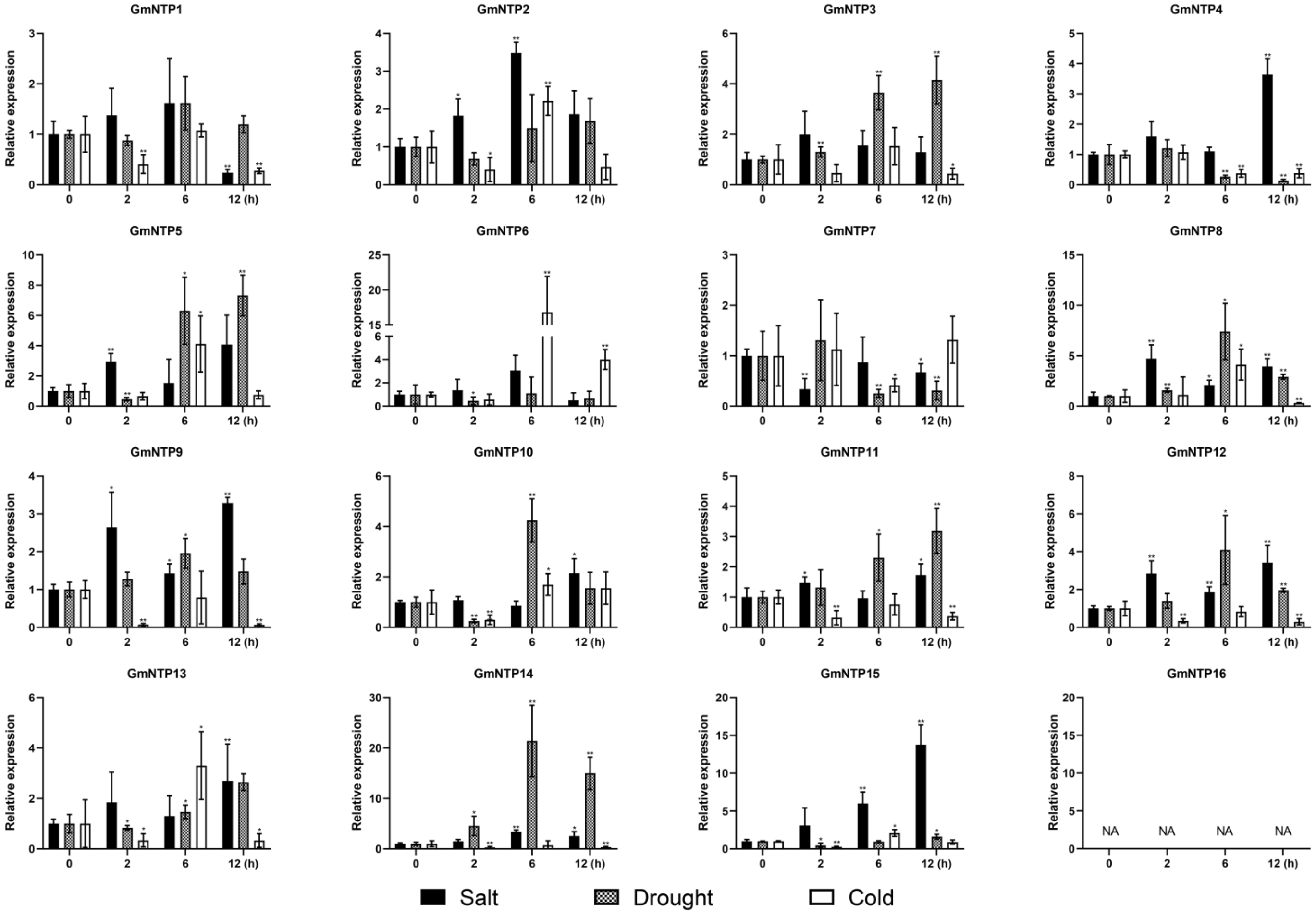

Figure S7. The differential expression analysis of GmNTP genes under salt, drought and low temperature stress. The bar graph illustrating the expression level changes of GmNTP genes identified by qRT-PCR under abiotic stress. The data are shown as the means ± SDs of three independent biological replicates. The different symbols above the bar chart indicate the level of significant difference, *, $P<0.05$; **, $p<0.01$.

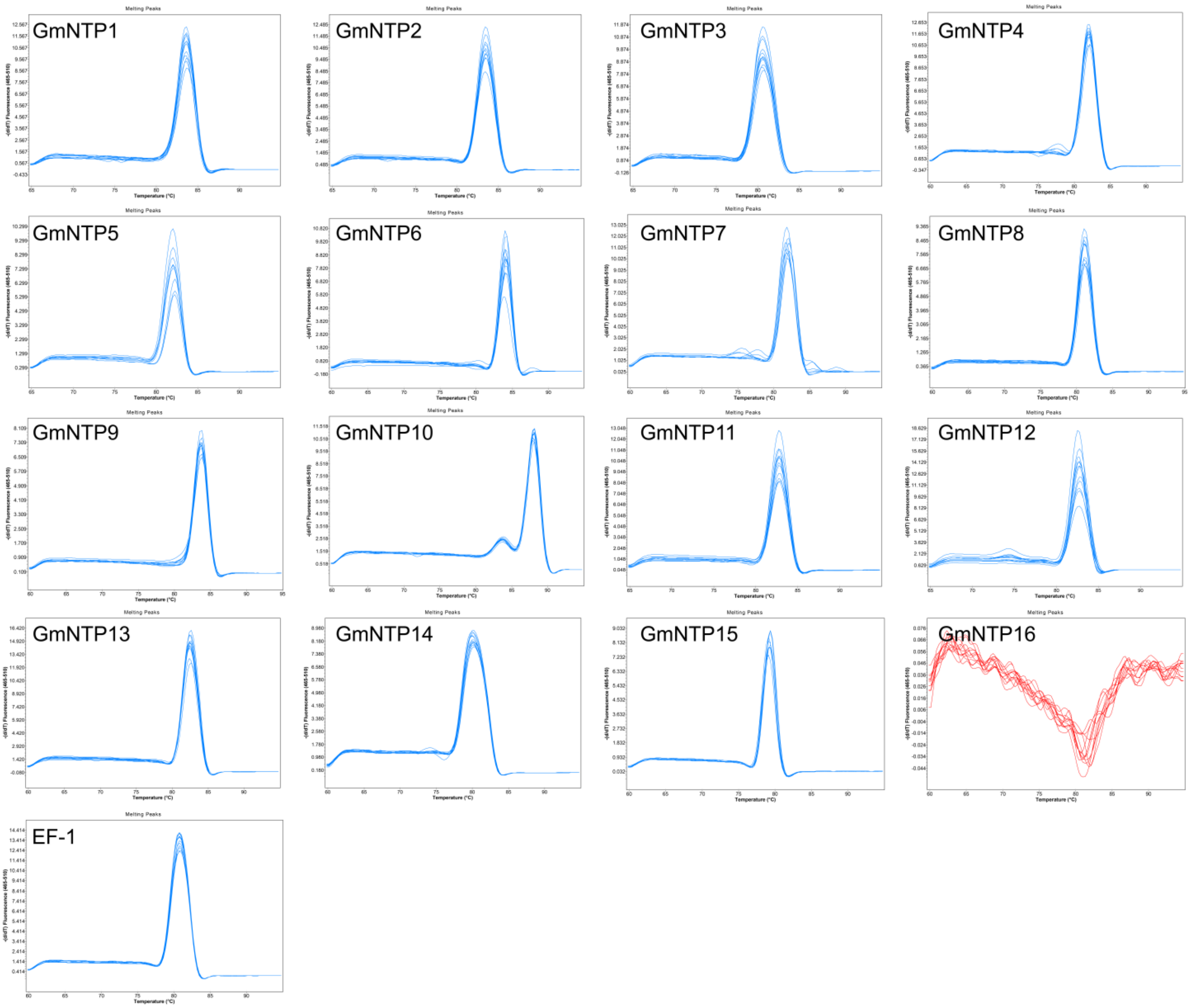

Figure S8. The melting peak was generated in qRT-PCR experiment, including reference gene and *GmNTP* genes.
